# Spatial single-cell isotope tracing reveals heterogeneity of de novo fatty acid synthesis in cancer

**DOI:** 10.1038/s42255-024-01118-4

**Published:** 2024-09-09

**Authors:** Elena Buglakova, Måns Ekelöf, Michaela Schwaiger-Haber, Lisa Schlicker, Martijn R. Molenaar, Mohammed Shahraz, Lachlan Stuart, Andreas Eisenbarth, Volker Hilsenstein, Gary J. Patti, Almut Schulze, Marteinn T. Snaebjornsson, Theodore Alexandrov

**Affiliations:** 1 Structural and Computational Biology Unit, European Molecular Biology Laboratory (EMBL), Heidelberg, Germany; 2 Cell Biology and Biophysics Unit, EMBL, Heidelberg, Germany; 3 Department of Chemistry, Washington University in St. Louis, St. Louis, MO USA; 4 Center for Metabolomics and Isotope Tracing, Washington University in St. Louis, St. Louis, MO USA; 5 Department of Medicine, Washington University in St. Louis, St. Louis, MO USA; 6 Division of Tumor Metabolism and Microenvironment, German Cancer Research Center (DKFZ) and DKFZ-ZMBH Alliance, Heidelberg, Germany; 7 Metabolomics Core Facility, EMBL, Heidelberg, Germany; 8 Molecular Medicine Partnership Unit, EMBL and Heidelberg University, Heidelberg, Germany; 9 BioStudio, BioInnovation Institute, Copenhagen, Denmark; 10 Department of Pharmacology, University of California San Diego, La Jolla, CA USA; 11 Department of Bioengineering, University of California San Diego, La Jolla, CA USA

**Keywords:** Metabolomics, Biological techniques

## Abstract

While heterogeneity is a key feature of cancer, understanding metabolic heterogeneity at the single-cell level remains a challenge. Here we present ^13^C-SpaceM, a method for spatial single-cell isotope tracing that extends the previously published SpaceM method with detection of ^13^C_6_-glucose-derived carbons in esterified fatty acids. We validated ^13^C-SpaceM on spatially heterogeneous models using liver cancer cells subjected to either normoxia-hypoxia or ATP citrate lyase depletion. This revealed substantial single-cell heterogeneity in labelling of the lipogenic acetyl-CoA pool and in relative fatty acid uptake versus synthesis hidden in bulk analyses. Analysing tumour-bearing brain tissue from mice fed a ^13^C_6_-glucose-containing diet, we found higher glucose-dependent synthesis of saturated fatty acids and increased elongation of essential fatty acids in tumours compared with healthy brains. Furthermore, our analysis uncovered spatial heterogeneity in lipogenic acetyl-CoA pool labelling in tumours. Our method enhances spatial probing of metabolic activities in single cells and tissues, providing insights into fatty acid metabolism in homoeostasis and disease.

## Abstract (web summary)

Buglakova et al. present ^13^C-SpaceM, a method that combines stable isotope tracing with imaging mass spectrometry thus enabling spatial analysis of lipid dynamics with near-single-cell resolution in tissues.

## Main

Lipids are a complex class of biomolecules involved in multiple cellular functions as structural components of cellular membranes, energy source and signalling molecules^1–5^. Altered lipid metabolism is a hallmark of cancer, as it supports rapid growth and survival and mediates oxidative stress resistance^6–9^, and is affected by nutrient and oxygen limitations in the tumour microenvironment^10^. Cancer cells may adopt different routes for lipid provision, thus inducing selective metabolic dependencies that could be targeted for cancer therapy.

**Fig. 1:**
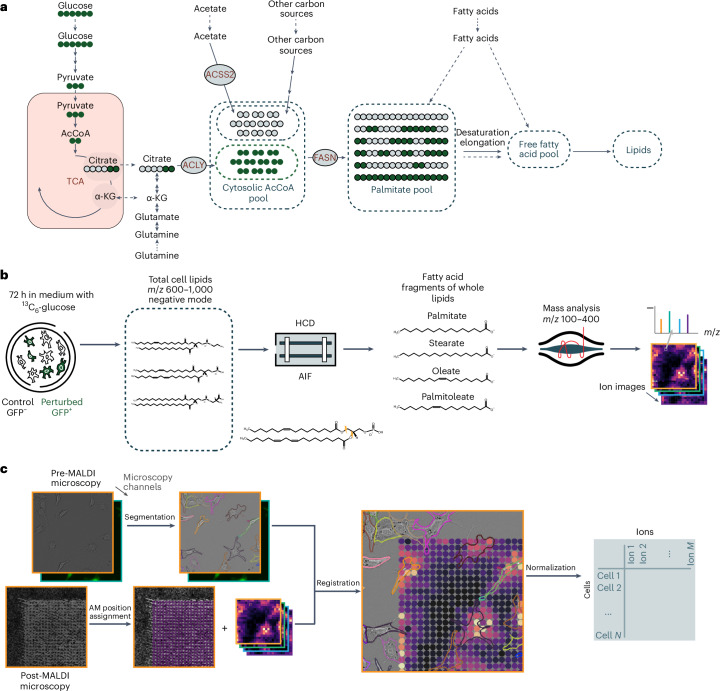
^13^C-SpaceM workflow as applied to interrogate de novo fatty acid synthesis. **a**, Generation of cytoplasmic acetyl-CoA (AcCoA) and subsequent fatty acid and lipid synthesis from stable isotope labelled ^13^C_6_-glucose. Glucose-derived pyruvate is metabolized in the mitochondria and exported to the cytoplasm in the form of citrate. ACLY converts citrate to acetyl-CoA, thereby contributing to the cytoplasmic (lipogenic) AcCoA pool. De novo fatty acid synthesis by fatty acid synthase (FASN) results in the formation of palmitate (C16:0), which is either further modified or directly incorporated into cellular lipids. Exogenously taken up fatty acids are also incorporated into cellular lipids but are not labelled. **b**, Using AIF imaging MS for ^13^C-SpaceM. Cells are grown in medium supplemented with uniformly labelled ^13^C_6_-glucose for 72 h. The combination of wide-range isolation of parent lipid ions followed by HCD fragmentation and selective isolation of fatty acid fragments allows high-sensitivity measurements of fatty acids incorporated into lipids, conceptually similar to bulk extractions followed by saponification but with the benefit of retained spatial information and the exclusion of free fatty acids. **c**, Integration of microscopy and imaging MS in ^13^C-SpaceM to obtain single-cell profiles. Pre-MALDI microscopy and post-MALDI microscopy images containing information about the cell outlines and areas ablated by MALDI imaging are registered. Ion intensities from MALDI imaging are assigned to single cells through a normalization procedure. AM, ablation mark; HCD, higher-energy collisional dissociation; α-KG, α-ketoglutarate.

Most lipids are synthesized from fatty acids (Fig. 1a) that differ in their length and degree of saturation, which is the number of double bonds in the hydrocarbon chain^11^. Fatty acid biosynthesis uses acetyl-CoA as a substrate to generate palmitate (16:0), a 16-carbon saturated fatty acid (SFA) that can be further elongated and/or desaturated and subsequently incorporated into the lipidome. Fatty acid desaturation is critical for cell survival, as the mono-unsaturated fatty acid (MUFA) oleate protects cancer cells from lipotoxicity and endoplasmic reticulum stress inhibits apoptosis and promotes ferroptosis resistance^12–17^. Hypoxic and Ras-transformed cells take up MUFA to support proliferation and survival^18^.

The cytoplasmic (lipogenic) acetyl-CoA pool used as substrate for fatty acid synthesis can be derived from different precursors. In normoxia, glucose-derived carbons are used by the tricarboxylic acid (TCA) cycle to produce citrate, which serves as a substrate for ATP citrate lyase (ACLY) to generate acetyl-CoA. Under hypoxic conditions, glutamine can substitute as a precursor for acetyl-CoA synthesis through reductive carboxylation of glutamate^19,20^. Furthermore, hypoxic cancer cells can also use acetate via acetyl-CoA synthetase 2 (ACSS2) to generate acetyl-CoA^21,22^. As ACLY and ACSS2 have also been found to provide substrates for histone acetylation^23,24^, modulation of acetyl-CoA metabolism can also affect gene expression programmes in cancer cells.

The advent of single-cell tools has enabled the analysis of individual cells within heterogenic populations^25^. Although single-cell transcriptomics and proteomics has provided insights into the heterogeneity of transcriptional and translational programmes, these methods are insufficient to reveal the dynamics of lipid metabolism. For example, expression of carnitine palmitoyltransferase 1 (CPT1) can support either catabolic or anabolic metabolic programmes in cancer cells^26^. Thus far, probing metabolism at the single-cell level has been challenging due to the lack of technologies available for single-cell metabolomics^27^. Different experimental methods have been applied to evaluate the heterogeneity of lipid metabolism in cancer cells, including metabolic sensors for malonyl-CoA^28^ or dendrimers to evaluate fatty acid uptake^29^. A recent study applied dielectric barrier discharge ionization on single cells to determine alterations in lipid metabolism upon ACLY inhibition in pancreatic cancer cells^30^. However, these methods do not provide sufficient molecular resolution to interrogate acetyl-CoA metabolism.

We previously developed a spatially resolved mass spectrometry (MS) pipeline for the detection of intracellular metabolites at a single-cell resolution^31^. Stable isotope tracing has emerged as a powerful approach to determine the contribution of different metabolic precursors to the cytoplasmic acetyl-CoA pool^19,21,32,33^. We now describe ^13^C-SpaceM, a single-cell method that combines stable isotope tracing with the detection of fatty acids derived from selected lipid classes in a spatially resolved manner, using matrix-assisted laser desorption/ionization (MALDI) in combination with all-ion fragmentation (AIF). Like in the original SpaceM method, we integrate MALDI and microscopy data and furthermore, perform mathematical modelling of the isotopologue distribution of the labelled fatty acids.

We applied this method to interrogate the effect of hypoxia or genetic depletion of ACLY on de novo fatty acid synthesis in murine liver cancer cells, revealing substantial heterogeneity in the contribution of glucose to the cytoplasmic acetyl-CoA pool at the single-cell level. We also applied the MALDI-AIF methodology together with mathematical modelling to interrogate tissue sections of mouse brains harbouring GL261 glioma cells at near-single-cell resolution. This analysis revealed substantial spatial heterogeneity in isotopologue distribution of de novo synthesized fatty acids, which provides a proxy for the labelling degree in the lipogenic acetyl-CoA pool. Overall, our method opens new avenues to interrogate spatial heterogeneity in fatty acid synthesis and acetyl-CoA metabolism in cancer cells.

## Results

### ^13^C-SpaceM resolves metabolic states of single cells

**Extended Data Fig. 1:**
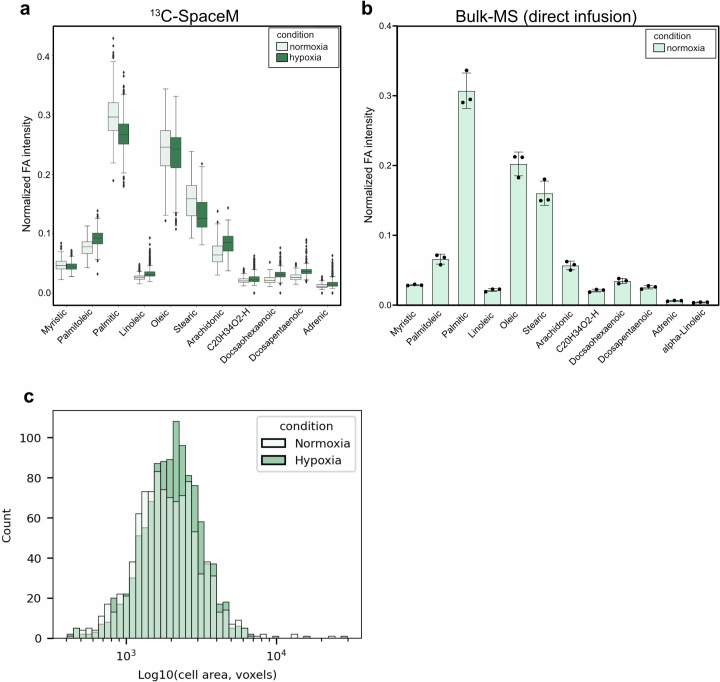
**a)** Box plots displaying mean and distribution of normalized intensities of 11 different fatty acids detected by 13C-SpaceM in normoxic and hypoxic cells. The box shows the quartiles of the dataset. Whiskers extend to show the range from Q1 – 1.5 IQR to Q3 + 1.5 IQR, where IQR is the inter quartile distance. The points that do not fall inside this range are considered to be outliers and are shown explicitly. **b)** Normalized intensities of 12 fatty acids released from total lipids isolated by saponification from normoxic cells and quantified using bulk MS with direct infusion. Error bars reflect standard deviation of the mean calculated from three biological replicates. **c)** Histogram of the distribution of cell area as determined by imaging for cells cultured in normoxia and hypoxia.

We developed ^13^C-SpaceM for single-cell isotope tracing into fatty acids. It builds upon SpaceM, a method for single-cell metabolomics integrating imaging MS and microscopy for assigning imaging MS pixels to individual cells and for quantifying fluorescence and morphometric properties of single cells^31^. To allow the selective interrogation of fatty acids esterified in cellular lipids, we included AIF-MS, where lipids in the range of 600–1,000 *m*/*z* ionized in negative mode were simultaneously fragmented and then the fragments in the range of 100–400 *m*/*z* were detected. The negative ion mode was chosen, as it allows detection of fatty acid RCOO^−^ ions. By performed imaging MS with the same mass range and ionization mode but without AIF, we detected a total of 64 lipid species from several lipid classes, including phosphatidic acids, phosphatidylinositols (PI), phosphatidylethanolamines (PE) and phosphatidylserines (PS). Peaks putatively annotated (corresponding to the Level 2 identification guidelines from the Metabolomics Standards Initiative) from these classes cover a majority of ions isolated in all experiments. The presence of the majority of these lipids was confirmed by bulk liquid chromatography (LC)–MS/MS (Supplementary Tables 1 and 2). The relative abundances of 11 abundant fatty acids identified using AIF-MS were closely matched by bulk MS analysis following saponification (Extended Data Fig. 1), confirming representative coverage. The MALDI-AIF imaging workflow allows the quantification of all isotopologues detected for fatty acids produced using AIF of lipids isolated at a fixed mass range of 600–1,000 *m*/*z* in the negative mode (Fig. 1b). Registration of microscopy images with MS images was conducted using SpaceM^31^ modified to include normalization and natural isotope abundance correction before the signal was assigned to individual cells (Fig. 1c).

**Fig. 2:**
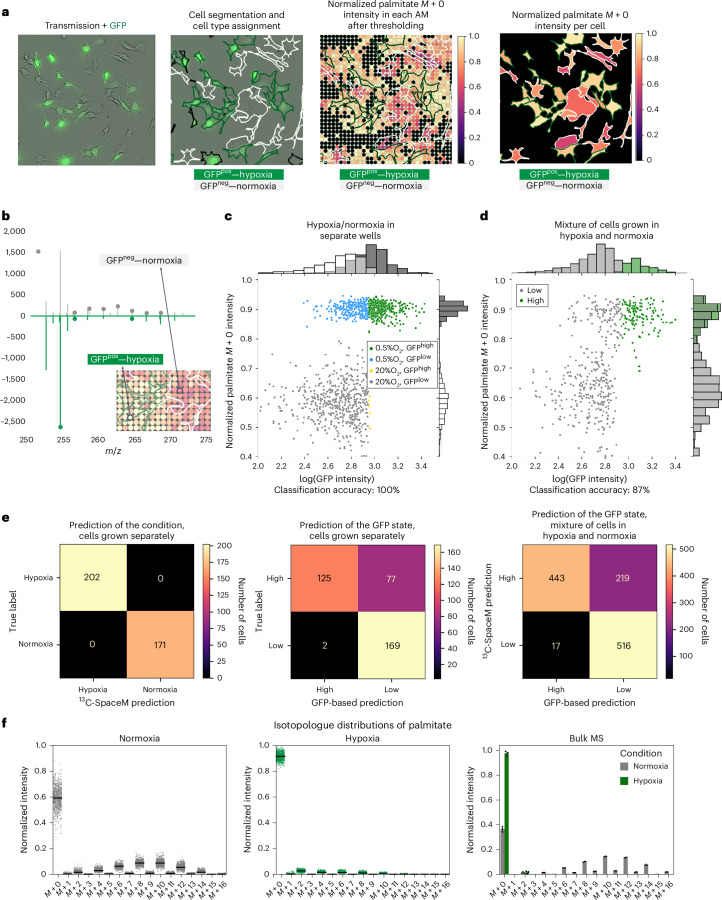
Validating ^13^C-SpaceM by interrogating de novo fatty acid synthesis in spatially heterogeneous normoxia-hypoxia model. **a**, Illustration of microscopy and imaging MS data from the model of co-plated primary murine liver cancer cells originally cultured under normoxia (GFP^neg^) and hypoxia (GFP^pos^). The GFP signal was used for discerning the culturing conditions (white and green cell outlines show normoxic and hypoxic cells, respectively). The normalized intensities of the M+0 isotope of palmitate (representing the fraction of unlabelled palmitate) are shown for MALDI imaging pixels and as assigned to the single cells. **b**, Mass spectra for individual pixels mapped to cells from a normoxic cell (white outline) and a hypoxic cell (green outline). The peaks corresponding to palmitate isotopologues are marked by grey or green dots. **c**, Discerning cells cultured under normoxia versus hypoxia using ^13^C-SpaceM for the cells mono-plated for each culturing condition. Scatter-plot and histograms show the values of the GFP reporter (ground truth for telling the condition) and the normalized intensity of the *M* + 0 isotope of palmitate representing the fraction of unlabelled palmitate for single cells. Different colours are used to show cells with different true state label, known from the growth conditions for the particular well, and different cell state assigned using GFP signal. Prediction accuracy refers to the prediction of the true state based on the isotopologue distributions. **d**, Same analysis as in **c** for spatially heterogeneous co-plated cells from both conditions. In this case accuracy refers to prediction of the cell state assigned using GFP signal. **e**, Confusion matrices for the prediction of culture condition based on ^13^C-SpaceM or GFP in cells grown separately and comparison of the two predictions for co-plated cells. **f**, Comparison of single-cell versus bulk intensities for the *M* + 0 isotope of palmitate. Single-cell intensities are from the spatially heterogeneous co-plated model. Bulk intensities are from cells of each mono-cultured condition subjected to total fatty acid analysis by saponification followed by LC–MS. For single-cell data, black lines show average values. For bulk data, data are displayed as mean ± s.d. across three replicates.

To validate ^13^C-SpaceM, we used a spatially heterogeneous model containing co-plated cells previously cultured under either normoxia (20% O_2_) or hypoxia (0.5% O_2_). As fatty acid synthesis exclusively utilizes the cytoplasmic acetyl-CoA pool, analysis of the isotopologue distribution of fatty acids allows the assessment of changes in the relative contribution of different substrates to the cytoplasmic acetyl-CoA pool^21^. Murine liver cancer cells were cultured in normoxia or hypoxia in medium containing U-^13^C-glucose for 72 h to achieve isotopic steady state for palmitate. Pre-MALDI microscopy images overlaying the brightfield and green fluorescent protein (GFP) channels for the normoxic (GFP^neg^) and hypoxic (GFP^pos^) cells demonstrated equal proportions of both cell populations (Fig. 2a, first panel). Cell segmentation and quantification of GFP provided single-cell fluorescence intensities (Fig. 2a, second panel). After applying an intensity threshold, the fraction of unlabelled palmitate (*M* + 0) was determined in a pixelated manner and calculated for single cells (Fig. 2a, third and fourth panels). Mass spectra collected at representative pixels from individual cells showed labelled palmitate isotopologues (*M* > 0) only in a normoxic cell, while the peak for unlabelled palmitate (*M* + 0) was predominant in the hypoxic cell (Fig. 2b). Thus, ^13^C-SpaceM was able to accurately measure differences in isotopologue patterns of palmitate in response to environmental perturbation at the single-cell level.

We also interrogated each condition separately by using mono-plated cultures. Figure 2c shows a single-cell scatter-plot displaying log (GFP intensity) as ground-truth readout characteristic of condition versus normalized palmitate *M* + 0 intensity calculated using single-cell isotopologue profiles. Cells cultured in hypoxia displayed normalized *M* + 0 intensities close to 0.9, indicating that only a small amount of palmitate was synthesized from glucose. This reflects either a low rate of de novo fatty acid synthesis or a switch to other substrates^21^. Normoxic cells showed lower normalized *M* + 0 intensities, with more heterogeneity compared with hypoxic cells (Fig. 2c). Heterogeneity could not be attributed to differences in cell size distribution (Extended Data Fig. 1c) or other morphometric parameters. In the co-plated model, cells assigned to the hypoxic condition (log_10_ GFP intensity >3) showed a large fraction of unlabelled palmitate (normalized *M* + 0 intensity around 0.9) (Fig. 2d), whereas cells with log_10_ GFP intensity <3 displayed a mixed phenotype (normalized *M* + 0 intensity between 0.4–0.8) (Fig. 2d). This was most likely caused by a proportion of hypoxic cells having lost GFP expression, potentially due to promoter silencing. Overall, we observed a strong similarity between mono- and co-plated cultures (Fig. 2c,d), although separation of the two populations was less obvious after co-plating. This could indicate an error due to co-sampling of neighbouring normoxic cells, causing a slight increase in intensities of labelled isotope peaks and reducing the unlabelled fraction. However, the presence of two distinct clusters indicates that this error was small, allowing a clear separation between the two phenotypes.

We quantified the capacity of ^13^C-SpaceM to discern the two phenotypes in an unbiased manner by assigning the conditions to single cells using GFP (log_10_ GFP intensity >3 indicating hypoxia). When levels of unlabelled palmitate were used as classifier, classification accuracy of 87% was achieved. We furthermore investigated the potential of ^13^C-SpaceM in predicting condition based on isotopologue profiles from other fatty acids (16:0, 16:1, 18:0, 18:1 and 14:0). Using a logistic regression classifier, we could predict the growth condition with perfect accuracy (Fig. 2e, left). In contrast, using GFP alone resulted in nearly 25% false negatives for the same population (Fig. 2e, middle), whereas the prediction based on ^13^C-SpaceM (Fig. 2e, right) returned a similar number of false negatives derived from GFP (22%). Overall, the high prediction accuracy of ^13^C-SpaceM validates the capacity of this method to identify changes in glucose contribution to palmitate and thus determine metabolic activity in a spatially heterogeneous model at single-cell resolution.

**Extended Data Fig. 2:**
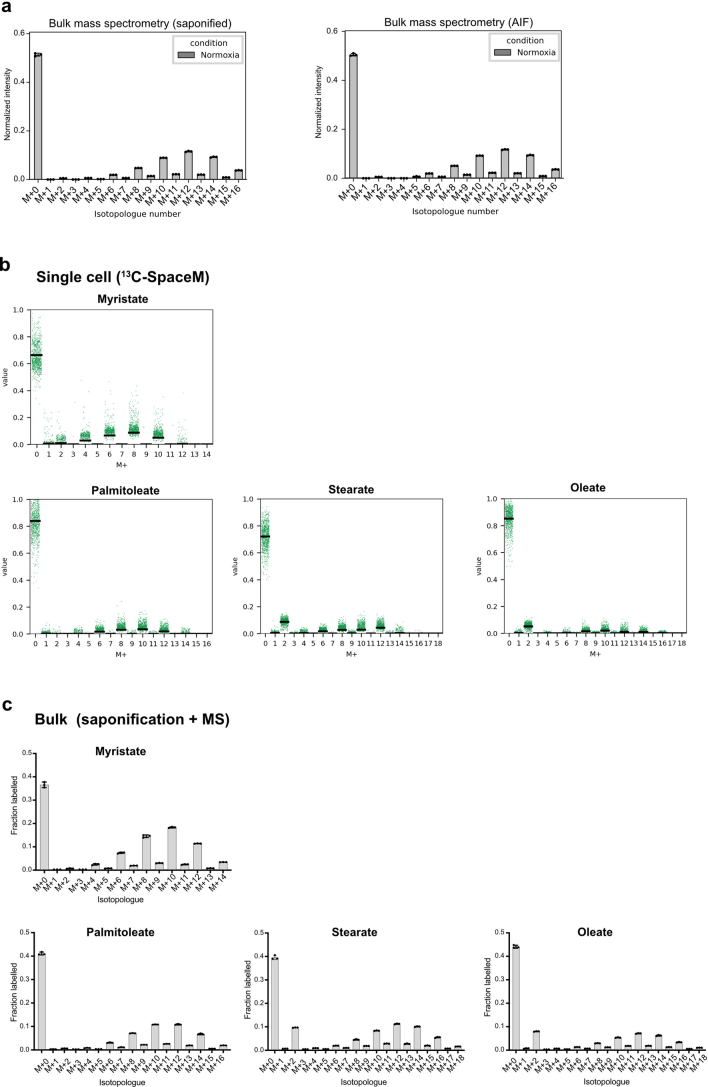
**a)** Comparison of isotopologue profiles of palmitate determined by bulk mass spectrometry after saponification (left graph) and all-ion fragmentation (right graph) from cells cultured in normoxia. Error bars reflect standard deviation of the mean calculated from three biological replicates. **b)** Single-cell isotopologue profiles for myristate, palmitoleate, stearate and oleate derived using ^13^C-SpaceM from cells cultured in normoxia. Black lines show average values. **c)** Isotopologue profiles for myristate, palmitoleate, stearate and oleate derived using bulk MS after saponification from cells cultured in normoxia. Error bars reflect standard deviation of the mean calculated from three biological replicates.

As additional validation, we compared single-cell isotopologue profiles with bulk measurements of lipids extracted from pooled cells, using a method for the isolation of major phospholipids, neutral lipids and ceramides^34,35^. Lipids were subjected to alkaline hydrolysis (saponification) to release fatty acids and palmitate isotopologue distribution was determined by LC–MS. Bulk analysis of palmitate showed an overall level of similarity with the pseudo-bulk data determined by ^13^C-SpaceM (Fig. 2f). However, there was a difference in the *M* + 0 fraction detected in normoxic cells between bulk and pseudo-bulk analysis, potentially due to chemical hydrolysis releasing fatty acids from a wider variety of lipids. We therefore applied AIF to bulk LC–MS analysis, using negative mode and mass range (600–1,000 *m*/*z*) as used in imaging MS (Extended Data Fig. 2a). This revealed a high similarity in palmitate isotopologue distribution between AIF and saponification, confirming that both methods interrogate the same lipid pools. We also compared isotopologue distribution of other fatty acids (myristate, palmitoleate, stearate and oleate) obtained by imaging MS with bulk analysis and found an overall concordance between the two methods (Extended Data Fig. 2b,c). Notably, the *M* + 2 isotopologue for stearate and oleate, generated by the elongation of unlabelled precursor, could be detected using both methods. The slightly lower intensity for the *M* + 0 isotopologues obtained using ^13^C-SpaceM could thus either be caused by differences in interrogated lipids (Supplementary Table 1) or underrepresentation of low abundance isotopologues due to lower sensitivity of imaging MS. Overall, we conclude that ^13^C-SpaceM can be used to determine glucose contribution to fatty acid synthesis by providing isotopologue distributions reflecting palmitate labelling as key readout of this pathway at single-cell level.

### Quantifying acetyl-CoA labelling degree in single cells

**Extended Data Fig. 3:**
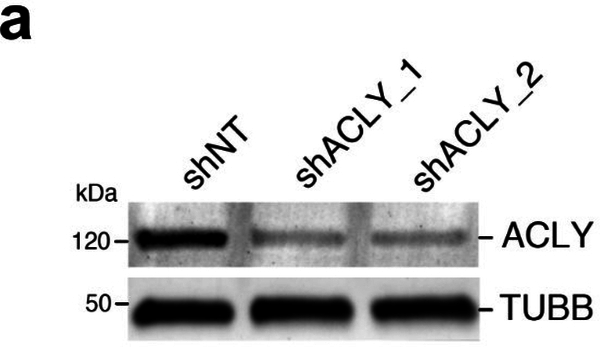
**a)** Immunoblot of protein lysates showing efficiency of ACLY knockdown using two shRNA sequences (ACLYkd oligo1 and ACLYkd oligo 2) compared to non-targeting control (shNT). Tubulin B (TUBB) is shown as loading control. This is an n = 1 western blot from a representative experiment using this knock down system for ACLY depletion. Source data

We next investigated the effect of genetically disrupting components of acetyl-CoA metabolism on fatty acid isotopologue profiles detected using ^13^C-SpaceM. ACLY catalysers the conversion of cytoplasmic citrate generated from glucose or glutamine into acetyl-CoA (Fig. 1a). We therefore engineered cells to express short hairpin RNAs (shRNA) targeting murine ACLY under the control of a doxycycline-inducible promoter. The same promoter also drives expression of a GFP reporter allowing identification of shRNA-expressing cells. We used two non-overlapping shRNA sequences (ACYL knockdown (ACLYkd) oligonucleotide (oligo) 1 and ACLYkd oligo 2) as well as a non-targeting control. Silencing was achieved by treating cells with 1 µg ml^−1^ of doxycycline for 72 h, with both shRNA sequences resulting in a comparable level of ACLY depletion (Extended Data Fig. 3a).

**Fig. 3:**
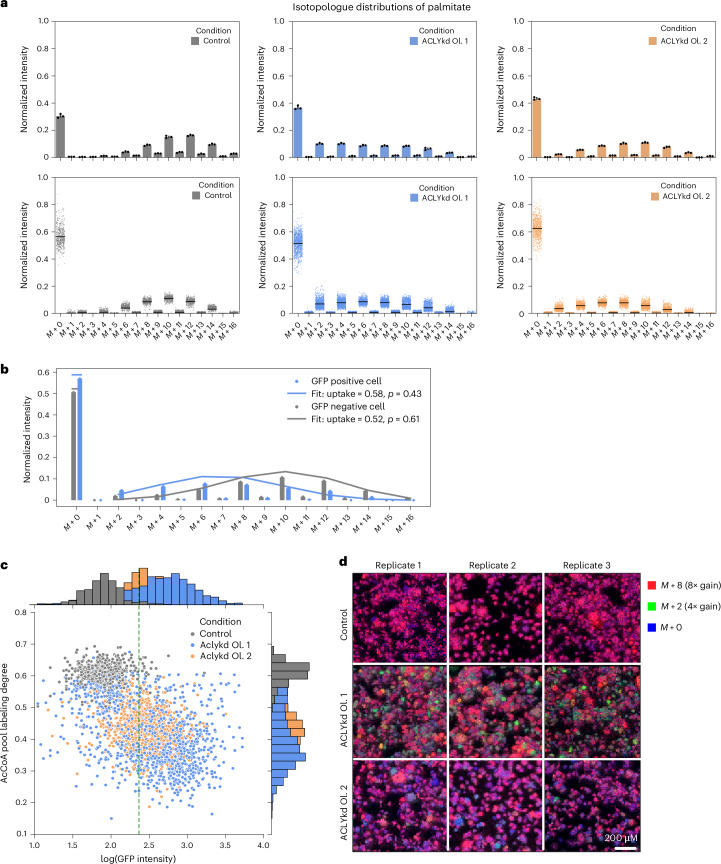
Single-cell quantitative analysis of lipogenic acetyl-CoA production and heterogeneity. **a**, Comparison of bulk (top) and single-cell (bottom) analysis of isotopologue distribution for palmitate after 72 h of ACLY gene silencing and culture in the presence of ^13^C_6_-glucose. Bulk data, generated by saponification and subsequent LC–MS, are displayed as mean ± s.d. across three replicates. For single-cell data, generated by MALDI with AIF, black lines show average values. **b**, Normalized single-cell isotopologue distributions for two individual cells, one from the control and the other from ACLYkd oligo 1 (shown as bar plots). Lines show fit of the fatty acid labelling binomial model: horizontal lines for *M* + 0 showing an estimated uptake and connected lines for *M* + 2*i* showing (1 − uptake) × binomial(*i*), where *i* is a variable from 1 to 8 indicating the even isotopic peaks. Legend shows parameters of the binomial model fit. **c**, Single-cell analysis of the estimated acetyl-CoA pool labelling degree (*p*) as calculated using the fatty acid labelling binominal model for the three conditions: control (grey), ACLYkd oligo 1 (blue) and ACLYkd oligo 2 (orange). Green dashed line shows 95% quantile of the GFP intensity distribution in the control condition, which was used to classify cells as GFP^pos^ versus GFP^neg^. **d**, Spatial metabolic imaging of de novo fatty synthesis for the control, ACLYkd oligo 1 and ACLYkd oligo 2 conditions. Abundance of different palmitate isotopologue peaks is displayed in different channels: *M* + 0 (blue), *M* + 2 (green) and *M* + 8 (red). Each channel is normalized to the TIC.

We induced ACLY silencing in cells cultured in the presence of U-^13^C-labelled glucose and interrogated them with ^13^C-SpaceM or bulk MS. Figure 3a shows isotopologue distribution for palmitate in the cells expressing non-targeting shRNA and after knockdown of ACLY induced by the two oligonucleotides. For the control cells, isotopologue distribution peaks at *M* + 10 in both bulk and single-cell analyses (Fig. 3a, left). Pseudo-bulk analysis of ^13^C-SpaceM data showing averages across all single cells indicates a higher *M* + 0 fraction compared with the bulk analysis (normalized peak intensity of 0.6 compared with 0.3). This is in line with the results for the normoxia-hypoxia model in Fig. 2f and, as discussed earlier, can be explained by differences in the specific lipid pools interrogated by the two methods. ACLY silencing using either oligo induced a marked shift in isotopologue distribution, with lower mass isotopologues being increased (Fig. 3a, middle and right). This is in line with our expectation that ACLY silencing reduces the contribution of glucose to the cytoplasmic acetyl-CoA pool.

The distribution of the pseudo-bulk intensities across the isotopologues detected after ACLY silencing using oligo 1 suggested the presence of two populations with distinct responses to gene silencing. We therefore used the power of single-cell analysis to deconvolve palmitate labelling in individual cells from the three conditions (control, ACLYkd oligo 1 and ACLYkd oligo 2). We calculated the acetyl-CoA pool labelling degree for each cell by applying a binomial model (described in Methods), with the estimated value *p* quantifying the fraction of labelled acetyl-CoA derived from glucose in the cytosolic acetyl-CoA pool. This model can be applied to each single-cell profile of isotopic intensities of any fatty acid by estimating *p*, which leads to best approximation of the data (the number of acetyl-CoA units, *n*, is equal to half the number of carbon atoms in each fatty acid, for example *n* = 8 for palmitate). Figure 3b shows examples of such modelling for two cells, a control cell (grey) and an ACLYkd oligo 2 cell (blue). As expected, the ACLYkd cell demonstrates a lower acetyl-CoA pool labelling degree (*p* = 0.43 versus 0.61).

Acetyl-CoA pool labelling degree (*p*) for all single cells in the three populations was plotted against the intensities of the GFP knockdown reporter (Fig. 3c). This showed a clear difference between the control cells and cells with ACLY knockdown, with knockdown resulting in lower values of *p* (histogram peak in control at 0.6 compared with 0.35 in ACLYkd oligo 1 and 0.4 in ACLYkd oligo 2). Moreover, the differences between the two ACLY knockdown conditions were also clearly visible. Oligo 1 resulted in a bimodal distribution of the values of *p*, with a visibly higher variance (values ranging from 0.2 to 0.65). A bimodal distribution in single-cell results often indicates the presence of two subpopulations. Notably, one mode of the distribution for the acetyl-CoA pool labelling degree in the ACLYkd oligo 1 condition displayed values for *p* in the range of 0.5–0.6 that overlapped with the values exhibited by control cells (*p* = 0.5–0.7). This can be interpreted as the presence of a subpopulation of ‘poorly silenced’ cells, where the knockdown was not sufficiently induced. Indeed, ACLYkd oligo 1 cells with high values of *p* (above 0.5) also display GFP intensities similar to those of wild-type cells, possibly due to the loss of the doxycycline-inducible expression cassette. The second mode of the distribution of the acetyl-CoA pool labelling degree (*p*) for ACLYkd oligo 1 cells had a much lower value (*p* = 0.35). This was clearly below the mode of the distribution of *p* for ACLYkd oligo 2 cells (*p* = 0.42). This indicates the presence of a ‘strongly silenced’ subpopulation of ACLYkd oligo 1 cells in which silencing was highly efficient, resulting in a strong reduction of acetyl-CoA labelling. This was contrasted by the results using oligo 2, where the acetyl-CoA pool labelling degree (*p*) as well as GFP reporter intensity displayed more homogenous values. Thus, ^13^C-SpaceM was able to detect heterogeneity in ACLY knockdown cells and identify different subpopulations.

Spatial information provided by ^13^C-SpaceM offers another view at cellular heterogeneity. Figure 3d shows ion images for three isotopologue peaks of palmitate (*M* + 0 in blue, *M* + 2 in green and *M* + 8 in red) for the three conditions (control, ACLYkd oligo 1 and ACLYkd oligo 2). The *M* + 2 peak was chosen as the most discriminative for the phenotype of low acetyl-CoA labelling specific to the ‘strongly silenced’ population among ACLYkd oligo 1 cells. The *M* + 8 peak was chosen as a representative for the control condition where fatty acids contain a high proportion of ^13^C. Thus, the difference between *M* + 2 and *M* + 8, as shown in Fig. 3a, can serve as an indicator of the relative contribution of glucose to the cytoplasmic acetyl-CoA pool and thus be used to display heterogeneity. The data were acquired with a pixel size of 10 µm with cells having an average area of 550 µm^2^, corresponding to 12 pixels per average cell. Ion images for ACLYkd oligo 1 showed a marked heterogeneity in the intensities of *M* + 2 and *M* + 8 isotopic peaks for palmitate that visualizes the presence of two distinct subpopulations in this condition. Notably, the observation that these two subpopulations were spatially heterogeneous ruled out a batch effect or technical artifacts and indicates a single-cell effect. In contrast, ACLYkd oligo 2 cells showed a more homogenous distribution of palmitate *M* + 8 and an overall lower abundance of the *M* + 2 peak. This degree of single-cell and spatial heterogeneity cannot be revealed through bulk analysis, demonstrating the unique advantages of the ^13^C-SpaceM method.

### ^13^C-SpaceM differentiates fatty acids by relative uptake

**Fig. 4:**
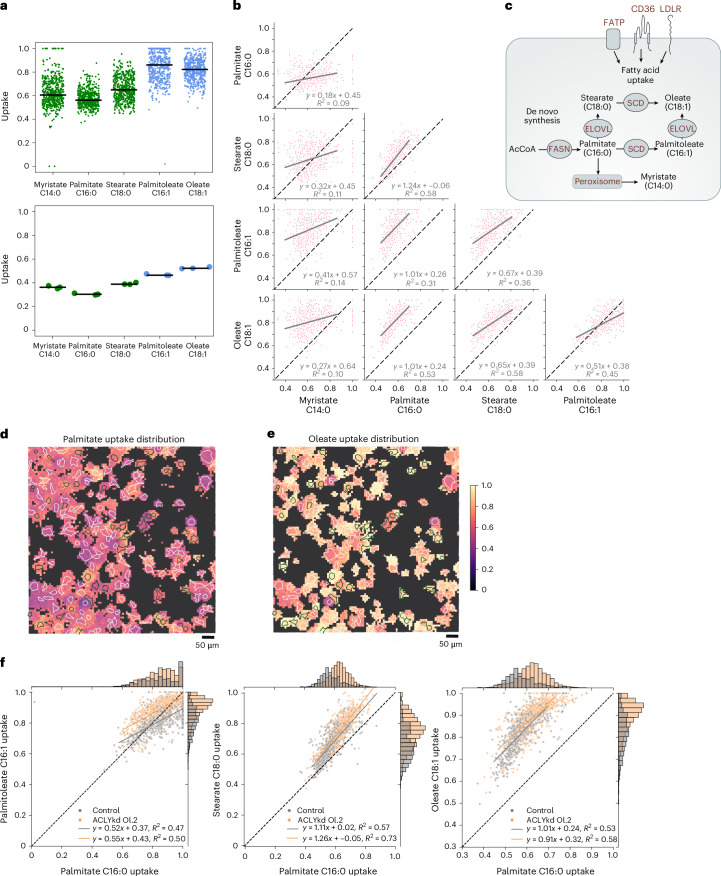
^13^C-SpaceM differentiates fatty acids by their relative uptake in single cells. **a**, Fatty acid uptake in the control cells as determined for myristate, palmitate, palmitoleate, stearate and oleate; SFAs in green and unsaturated fatty acids in blue. Single-cell data with each point representing a cell, black horizontal lines showing median values (top). Data from bulk stable isotope tracing (bottom). **b**, Correlation of uptake of different fatty acids. Dashed line represents the diagonal. For each pair of fatty acids, the linear fit is shown as a grey line, with the fit parameters shown in the legend. **c**, Diagram showing mechanisms involved in fatty acid synthesis, desaturation and uptake. **d**, Spatial metabolic imaging of the palmitate uptake (normalized *M* + 0 fraction) in co-plated cells from control and ACLYkd oligo 2 populations. Each square corresponds to one MALDI pixel. Pixels with the total intensity of the fatty acid isotopes below the set limit of detection (1,000) are shown in black. Cell outlines are green for GFP^pos^ cells (control) and white for GFP^neg^ cells (Aclykd.oligo2). Pixels are coloured according to the normalized *M* + 0 describing the uptake contribution for a given fatty acid. Image shows representative section of one out of six replicates. **e**, Same analysis as in **d** for oleate. **f**, Single-cell analysis of changes in fatty acid uptake upon ACLY knockdown for palmitate, palmitoleate, stearate and oleate. Dashed lines are the diagonal. For each condition the linear fit is shown in the corresponding colour, with the fit parameters shown in the legend. FATP, fatty acid transport proteins; LDLR, low density lipoprotein receptor; SCD, stearoyl-CoA desaturase.

We next considered that when isotopic steady state for a given fatty acid pool has been reached and the labelling degree of lipogenic acetyl-CoA is sufficient (distribution of labelled isotopologues does not overlap with *M* + 0), ^13^C-SpaceM data could be used to determine the relative uptake of different fatty acids compared with their de novo synthesis at a single-cell level. For each fatty acid, this was achieved by first performing natural isotope correction, normalizing all isotopologue peaks to the sum of all peaks, approximating isotopologue intensities with the binomial model described in Methods, and taking the value of the ‘uptake’ from the model. Figure 4a, top, shows single-cell values of the relative uptake for the detected fatty acids: SFAs myristate, palmitate, stearate as well as MUFAs palmitoleate and oleate, as determined in the control liver cancer cells. The levels of relative uptake at 60–90% (and thus de novo synthesis of 10–40%) are close to the reported rate of 30% of de novo lipogenesis in patients with non-alcoholic fatty liver disease^36^. The two MUFAs, palmitoleate and oleate, showed a substantially higher relative uptake compared with the corresponding SFAs palmitate and stearate. This indicates that these cells mostly utilize uptake rather than de novo synthesis to obtain MUFAs. The results on the average levels were confirmed in the bulk LC–MS-based isotope tracing (Fig. 4a, bottom).

We next investigated the relationships between the relative uptake of different fatty acids in the same cells. This was conducted by plotting and analysing the single-cell values of relative uptake for each fatty acid versus the others. This analysis showed no correlation of the relative uptake of myristate with any of the other fatty acids (Fig. 4b, first column). This lack of correlation is expected, as myristate was reported to be produced by de novo FA synthesis only to a small degree. Instead, myristate is mainly generated by the shortening of palmitate via peroxisomal β-oxidation or the elongation of lauric acid^37^. In contrast, single-cell relative uptake values for palmitate, palmitoleate, stearate and oleate showed strong positive correlations (Fig. 4b, columns 2–4). Thus, we can differentiate fatty acids by their sources specifically by exogenous uptake versus de novo synthesis. The lack of correlation for the single-cell uptake values for myristate likely reflects the specific mechanism of production or uptake of this fatty acid. Together, these data show that ^13^C-SpaceM can be used to determine relative uptake versus synthesis of different fatty acids at the single-cell level (Fig. 4c). However, it should be noted that this estimation may only apply to the lipid pools queried by the MALDI imaging MS method used here, as the relative contribution of fatty acids that are derived from uptake may well differ between lipid classes and/or pools.

Next, we investigated the effect of ACLYkd on fatty acid uptake. Spatial analysis and visualization demonstrated a strong single-cell heterogeneity in the relative uptake of either palmitate or oleate in a mixed population of control and ACLYkd oligo 2 cells (Fig. 4d,e). More detailed and quantified analysis, by plotting the single-cell relative uptake values separately for control and ACLYkd oligo 2, showed that ACLY-silenced cells increased the uptake of palmitate (histogram peak at 0.55 in control compared with 0.61 in ACLYkd), with each population displaying a unimodal symmetric distribution (Fig. 4f). Oleate uptake, which was already higher compared with palmitate uptake in the control cells, was also increased after ACLY knockdown (histogram peak at 0.85 in control compared with 0.95 in ACLYkd). In addition, we observed an asymmetric (skewed) unimodal distribution of the oleate single-cell relative uptake values for the ACLYkd population, with more cells showing high relative uptake values. This indicates that some cells of the population respond to ACLY silencing by selectively inducing the uptake of MUFAs, such as oleate.

### Tissue heterogeneity of fatty acid and acetyl-CoA synthesis

Our results have shown that combining MALDI imaging MS with AIF to detect esterified fatty acids instead of the lower-abundant free fatty acids substantially increases sensitivity and allows the analysis of isotopologue distribution to assess cytoplasmic acetyl-CoA labelling at the level of single cells. This prompted us to apply this methodology to tumour tissue sections at a near single-cell spatial resolution, to investigate whether metabolic constraints imposed by the tumour microenvironment, such as differential access to nutrients and/or oxygen result in intra-tumoural heterogeneity in terms of glucose contribution to the cytosolic acetyl-CoA pool or the relative contribution of fatty acid uptake versus de novo synthesis. We analysed brain tissue sections from mice that had been orthotopically implanted with GL261 glioma cells expressing mutant isocitrate dehydrogenase 1 (IDH1) and red fluorescent protein (RFP). The mice were fed either an unlabelled or U-^13^C-glucose-containing liquid diet for 48 h before tissue collection. Sections from the same tissue samples had been analysed previously using MALDI- and DESI-imaging MS to study the incorporation of glucose-derived carbons into various different metabolites including free (non-esterified) fatty acids^38^. That analysis revealed spatial differences in isotopologue distribution of free palmitate and stearate between tumour and non-tumour regions of the brain, but did not provide sufficient spatial resolution to fully appreciate intra-tumoural heterogeneity^38^.

**Fig. 5:**
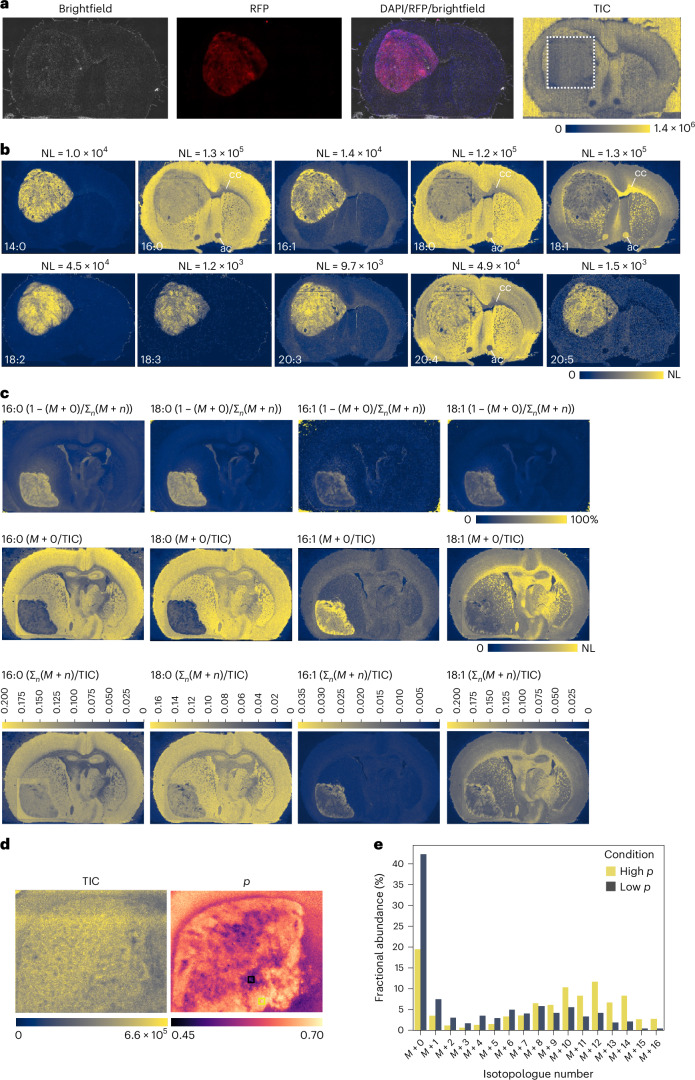
^13^C-SpaceM reveals intra-tumoural heterogeneity of fatty acid synthesis at a near-single-cell resolution. **a**, Left to right: brightfield image of a cryosection from the brain of a tumour-bearing mouse after orthotopic implantation of GL261 glioma cells. Visualization of RFP-expressing glioma cells in the brain. Staining of the cryosection with 4,6-diamidino-2-phenylindole (DAPI) overlapped with the RFP and brightfield channels. Visualization of TIC obtained by MALDI imaging MS and AIF. Square denotes a region where data were acquired at a near-single-cell resolution (10 µm pitch as opposed to 50 µm pitch for the rest of the section). Scale bar ends at the highest point of intensity in the image. **b**, Visualization of the localization of ten different esterified fatty acids in a section from a tumour-bearing brain. NL value (highest signal intensity in the image) for each fatty acid is shown above the image. ac, anterior commissure; cc, corpus callosum. **c**, Fractions of palmitate, palmitoleate, stearate and oleate derived from fatty acid synthesis during the labelling period (48 h) visualized by displaying the sum of all labelled isotopologues as a fraction of the sum of all isotopologues (1 − (*M* + 0)/Σ_*n*_(*M* + *n*)) (top). The unlabelled isotopologue (*M* + 0) for palmitate, palmitoleate, stearate and oleate normalized to the TIC (middle). Sum of all isotopologues shown as a fraction of the TIC; scale bar shows fraction of TIC (bottom). **d**, Visualization of the TIC and the degree of labelling of the cytoplasmic acetyl-CoA pool (*p*) in the tumour. **e**, Isotopologue distributions derived from a high and a low *p* area of the tumour (marked by yellow and black squares in **d**, respectively).

We first applied our methodology to analyse the esterified fatty acid composition in brain sections from tumour-bearing mice that were fed a ^12^C-glucose diet. Comparison of the total ion count (TIC) with brightfield and fluorescent imaging revealed high ion counts throughout the whole brain, including the tumour area (Fig. 5a). The tumour area (Fig. 5a, TIC plot, boxed) was analysed using a higher resolution (10 µm pixel size) compared with the rest of the tissue section (50 µm). Spatial analysis of different fatty acids revealed a high amount of heterogeneity in fatty acid abundances in the non-tumour-bearing hemisphere, with individual structures such as the corpus callosum and anterior commissure identified based on their fatty acid composition alone, with both regions being high in oleate (18:1) and low in palmitate (16:0), stearate (18:0) and arachidonate (20:4) relative to surrounding brain tissue (Fig. 5b). While palmitate, stearate, oleate and arachidonate were present at similar levels both in tumours and the surrounding brain, myristate (14:0) and palmitoleate (16:1) were substantially increased in the tumour tissue. Notably, the essential fatty acids linoleate (18:2) and α/γ-linolenate (18:3) were also selectively higher in the tumour compared with the rest of the brain tissue (Fig. 5b).

**Extended Data Fig. 4:**
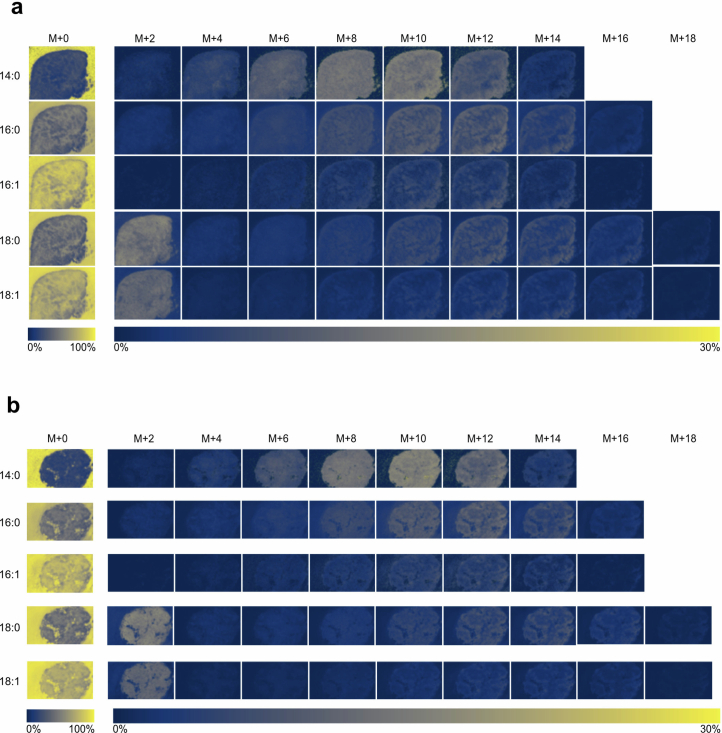
**a)** Isotopologue fractional images for myristate (14:0), palmitate (16:0), palmitoleate (16:1), stearate (18:0) and oleate (18:1) in tumour tissue (tumour #1) from a mouse orthotopically implanted with GL261 IDH1 mutant glioma cells and fed with a liquid diet containing U-^13^C_6_-Glucose for 48 hours. Colours represent fraction of each isotopologue of the sum of all isotopologues for each fatty acid. **b)** Isotopologue fractional images for myristate (14:0), palmitate (16:0), palmitoleate (16:1), stearate (18:0) and oleate (18:1) in tumour tissue (tumour #2) from a mouse orthotopically implanted with GL261 glioma cells and fed with a liquid diet containing U-^13^C_6_-Glucose for 48 hours. Colours represent fraction of each isotopologue of the sum of the all isotopologues for each fatty acid.

We next analysed tissues from tumour-bearing mice that had been fed a diet containing U-^13^C-glucose for 48 h and determined the isotopologue distribution of the five major esterified fatty acids derived from de novo fatty acid synthesis selectively in the tumour tissue (Fig. 5c and Extended Data Fig. 4a,b). The three SFAs, myristate (14:0), palmitate (16:0) and stearate (18:0), showed a high relative abundance of ^13^C incorporation, with isotopologue distributions peaking at *M* + 10, *M* + 12 and *M* + 14, respectively (Extended Data Fig. 4a,b). These results suggest that myristate was almost exclusively derived from de novo fatty acid synthesis, as the intensity of *M* + 0 was very low compared with the other isotopologues. As myristate is important for the post-translational modification of important signalling proteins^39^, this finding suggests that glioma tumours may selectively upregulate myristate synthesis to promote their growth. In contrast, the two MUFAs, palmitoleate (16:1) and oleate (18:1), showed a higher relative abundance of the *M* + 0 isotopologue (Extended Data Fig. 4a,b). In addition, stearate and oleate exhibited a pronounced abundance of the *M* + 2 isotopologue, indicative of elongation from unlabelled precursors (palmitate and palmitoleate, respectively). This was in line with the previous analysis demonstrating prominent fatty acid elongase activity in these tumours^38^.

To analyse this in more detail, we calculated the fraction of all ^13^C isotopologues of the total $1-(M+0)/{\sum }_{n}(M+n)$, representing the fraction of the respective fatty acid that was derived from de novo synthesis. This revealed that the majority of palmitate in the tumour was derived from de novo fatty acid synthesis within the timeframe of the ^13^C labelling (Fig. 5c). In contrast, the fraction of palmitoleate derived from de novo fatty acid synthesis was lower (Fig. 5c), despite palmitoleate being enriched in the tumour tissue (Fig. 5b). Oleate showed a higher labelling degree than palmitoleate (Fig. 5c), but this was mostly linked to the high proportion of the *M* + 2 isotopologue (Fig. 5c and Extended Data Fig. 4a,b), which is formed by elongation of unlabelled palmitoleate. Considering that the tumour-bearing mice were fed a ^13^C-glucose diet for only 48 h^38^, it is likely that isotopic steady state for these fatty acids was not reached and thus any exact quantification of the contribution of de novo synthesis or uptake in the tumour is not possible. Nevertheless, we found that the pool sizes for palmitate, stearate and oleate were in a similar range, whereas the pool size of palmitoleate was far smaller (Fig. 5c, bottom row). Thus, the low fraction of palmitoleate and oleate derived from de novo synthesis was not due to their pool sizes being larger relative to that of their precursors. This indicates that these fatty acids are not derived from de novo synthesis within the timeframe of the labelling but rather originate from uptake. These findings suggest that tumours have limited Δ9-desaturase activity, provided by stearoyl-CoA desaturase and rely on the microenvironment for the provision of MUFAs.

We next used the isotopologue distribution of palmitate to calculate the fraction of the cytosolic acetyl-CoA pool derived from glucose on a pixel-by-pixel basis, similar to the in vitro analysis (Fig. 3c), to generate a spatial representation of acetyl-CoA labelling in the tumour. This revealed a significant degree of spatial intra-tumoural heterogeneity, with glucose contribution to the cytosolic acetyl-CoA pool (*p*) ranging from 0.45 to 0.70 (Fig. 5d). It should be noted that the observed variability in *p* was not due to differences in TIC, which was quite uniform across the sample (Fig. 5d). Close inspection of selected areas with high and low *p* further highlighted the differences in palmitate isotopologue distribution (Fig. 5e). Thus, MALDI imaging MS coupled with AIF is capable of determining the labelling degree of the cytosolic acetyl-CoA pool to visualize spatial intra-tumoural metabolic heterogeneity in tissue sections at near-single-cell resolution.

### Characterizing essential fatty acid metabolism in glioma

**Fig. 6:**
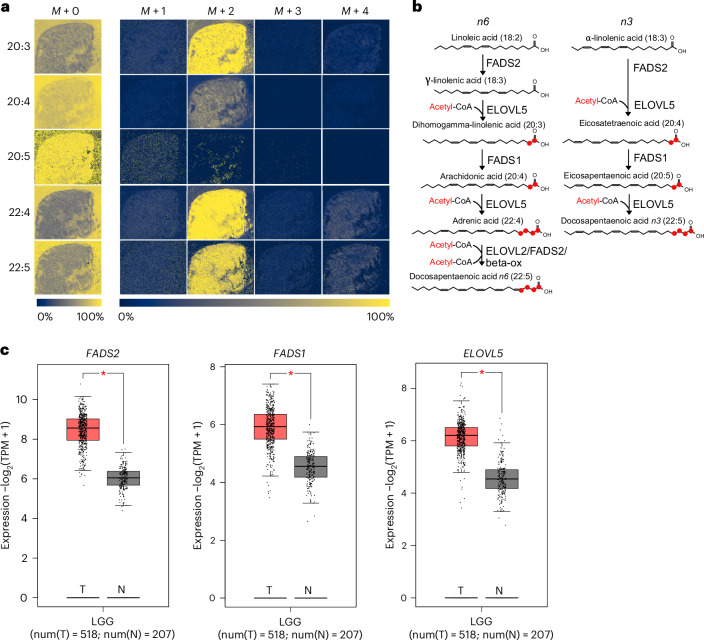
Glioma tumours differentially process *n3* and *n6* essential fatty acids. **a**, Isotopologue distribution for the polyunsaturated fatty acids 20:3, 20:4, 20:5, 22:4 and 22:5. Scale bar shows the fraction of each isotopologue from the sum of all isotopologues for each fatty acid. **b**, Schematic of the omega-3 and omega-6 polyunsaturated fatty acids biosynthesis pathways. Carbon atoms added from acetyl-CoA by fatty acid elongation are indicated in red. **c**, Comparison of RNA expression for enzymes involved in the processing of essential fatty acids, FADS1, FADS2 and ELOVL5 in low-grade glioma (LGG) samples from The Cancer Genome Atlas (TCGA) (*n* = 518) and corresponding normal brain (from the Genotype-Tissue Expression (GTEx) project) (*n* = 207). Analysis was performed using GEPIA2 with a *P* value cutoff of 0.01 (in all cases (indicated by a single asterisk), the *P* values were below this cutoff) using one-way analysis of variance and the results presented as a boxplot.

Our finding that the essential fatty acids linoleate (18:2) and α/γ-linolenate (18:3) accumulate exclusively in the tumour (Fig. 5b) raised the question whether these fatty acids are also processed further, as their derivatives serve as precursors for the synthesis of lipid mediators, such as prostaglandins and leukotrienes that play important roles in cancer^3^. We therefore analysed the specific isotopologue distributions of several derivatives of essential fatty acids, namely 20:3, 20:4, 20:5, 22:4 and 22:5 (Fig. 6a). We found that the *M* + 2 isotopologue fractions of all except 20:5 were enriched in the tumours (the pool size for these fatty acids was either similar or higher in the tumour region relative to the brain, which excludes the possibility of this being an artifact of not having reached isotopic steady state). This suggests that glioma tumours not only take up essential fatty acids from the microenvironment but also process these further by elongation and desaturation. The enzymes required for essential fatty acid metabolism, fatty acid elongase 5 (ELOVL5), fatty acid desaturase 1 (FADS1) and fatty acid desaturase 2 (FADS2) are upregulated in low-grade glioma (Fig. 6b,c). Our data also suggest differences in the processing of omega-3 and omega-6 essential fatty acids, as eicosapentaenoic acid (20:5), a PUFA exclusive to the omega-3 branch (Fig. 6b), shows no label incorporation (Fig. 6a). This suggests that the tumour mostly engages in the processing of omega-6 essential fatty acids, which can give rise to pro-inflammatory lipid mediators, such as prostaglandin E2 (PGE_2_).

Taken together, we have presented ^13^C-SpaceM, a sensitive and robust method for spatial isotope tracing into esterified fatty acids in single cells, and used a similar methodology to analyse tissue sections at near single-cell resolution. We demonstrated how this method can be used to study fatty acid uptake, acetyl-CoA metabolism, and activity of de novo fatty acid synthesis in single cells. In tissue sections of mouse glioma, we could show spatial intra-tumour heterogeneity of acetyl-CoA synthesis. The novelty of this method lies in employing AIF-MS combined with mathematical modelling for isotopologue analysis of labelled fatty acids. Using AIF helps to enhance the sensitivity of the analysis at high spatial resolution, while mathematical modelling provides quantitative information on lipogenic acetyl-CoA pool labelling. Our development addresses the need to investigate the activity of metabolic pathways by resolving single-cell and spatial heterogeneity with high molecular sensitivity.

## Discussion

The advance of spatial and single-cell -omics technologies has revolutionized biology and provided insights into the genetic and phenotypic heterogeneity of cell populations at the levels of tissues, organs and whole organisms^25^. This is of particular importance in cancer, as tumours consist of multiple sub-clones of cancer cells as well as a highly diverse population of stromal cells that can change in a spatial and temporal manner^40^. Complementing the paradigm of genetic heterogeneity^41^, the concept of metabolic heterogeneity and plasticity in cancer is emerging^42–44^, particularly as the metabolic state of cancer cells is influenced by non-genetic factors, including nutrients and oxygen in the tumour microenvironment^42^. Recent technological advances in MS, including electrospray ionization (ESI), MALDI or dielectric barrier discharge ionization in combination with advanced computational methods enabled the detection of polar metabolites and lipids from single cells^30,31,45^. While these methods have provided unprecedented insight into the metabolic states of single cells, they fail to capture dynamic metabolic processes or flexibility of the metabolic network. Stable isotope tracing has emerged as gold-standard to reveal metabolic activity in cells and tissues in bulk^46^ and in a spatially resolved manner^38,47^, but was so far not demonstrated in single cells, mostly due to sensitivity limits.

We developed ^13^C-SpaceM, a method to combine stable isotope tracing with spatial single-cell metabolomics to monitor de novo fatty acid biosynthesis at single-cell resolution. ^13^C-SpaceM builds upon the previously published SpaceM method^31^ with several modifications, including additional data analysis for the quantification of multiple isotopologues of fatty acids. In particular, we included a binomial model to determine the labelling degree of the lipogenic acetyl-CoA pool from fatty acid isotopologue data for individual cells^48^. The use of AIF to release esterified fatty acids results in high sensitivity and resolution. Nevertheless, this largely restricts our analysis to fatty acids released from a subset of lipid classes that are effectively ionized in negative mode, primarily acidic glycerophospholipids. Despite these limitations, this approach is sufficient to determine the contribution of a labelled substrate (for example glucose) into the lipogenic acetyl-CoA pool based on isotopologue distribution of fatty acids, which was a major aim of this study.

We validated the ability of ^13^C-SpaceM to detect changes in glucose-dependent fatty acid synthesis at single-cell resolution by co-plating cells from different metabolic states (hypoxia/normoxia). This model was chosen as hypoxia leads to a global reprogramming of lipid metabolism^10^. Spatial single-cell isotope tracing by ^13^C-SpaceM clearly separated the two cell populations based on the strong increase in palmitate *M* + 0 fraction in hypoxic cells. This is caused by inhibition of pyruvate dehydrogenase^49^, leading to reduced flux of glucose carbons into the TCA cycle and subsequently the lipogenic acetyl-CoA pool. Hypoxia also promotes the reductive carboxylation of glutamine^19,20^ and synthesis of acetyl-CoA from acetate^21,22^. In addition, hypoxia reduces fatty acid synthesis and stimulates fatty acid uptake^18,50^ which also increases the proportion of the palmitate *M* + 0 fraction.

We also assessed the application of ^13^C-SpaceM to monitor the effect of genetic perturbations of the metabolic network. Bulk and single-cell analyses revealed a marked shift in palmitate isotopologue distribution following silencing of ACLY, the enzyme catalysing the conversion of citrate to acetyl-CoA. Using a binomial model to quantify fractional labelling from the isotopologue distribution^21^, we estimated the labelling degree of acetyl-CoA in control and ACLY-silenced single cells. In agreement with previous studies using bulk analysis in ACLY wild-type versus knockout mouse embryo fibroblasts^23^, silencing of ACLY caused a reduction in the fractional labelling of acetyl-CoA from glucose but only a minor increase in the *M* + 0 fraction of palmitate. This suggests that other substrates, such as acetate, are used to synthesize acetyl-CoA when ACLY is absent^23^. We also revealed substantial heterogeneity in fractional labelling of lipogenic acetyl-CoA within the ACLY-silenced cell populations, suggesting differences in efficiency of the shRNA sequences completely obscured in the bulk analysis. As acetyl-CoA is also a substrate for protein post-translational modification, methods to quantify acetyl-CoA synthesis from different substrates at single-cell level can provide insight into genetic and environmental factors that drive heterogeneity in cancer.

Fatty acid uptake via the scavenging receptor CD36 has been shown to facilitate metastasis formation and stem cell phenotypes in cancer^51^^,52^. We therefore used ^13^C-SpaceM to determine the relative proportion of de novo synthesis and uptake of five non-essential fatty acids at single-cell level. This revealed substantial heterogeneity in the relative uptake of different fatty acids, with a higher proportion of uptake for palmitoleate and oleate compared with palmitate, suggesting high demand for MUFAs. Moreover, ACLY silencing caused a general shift from de novo synthesis to uptake of palmitate and oleate, consistent with a recent single-cell lipidomics study observing reduced levels of PC species containing saturated and MUFAs upon chemical inhibition of ACLY in pancreatic cancer cells^30^.

We also applied our methodology to brain sections from mice bearing glioma xenografts, enabling the visualization of different esterified fatty acids in tumour and non-tumour tissue. This revealed highly selective partitioning of individual fatty acid species to specific brain structures, which could be further explored by integrating our results with spatial transcriptomics data from the brain atlas^26^ or future correlative studies combining imaging MS and other spatial omics techniques. We also used tissue from mice fed a liquid diet containing U-^13^C-glucose for 48 h to achieve deep labelling of the entire metabolic network, including lipids^53^. Isotopologue analysis revealed a strong induction of synthesis of SFAs, myristate, palmitate and stearate, in tumour tissue compared with the surrounding brain, thus confirming previous results obtained in the same model^38^. This is in agreement with previous studies indicating that fatty acid synthesis is required for brain metastasis in breast and other cancers^54,55^. However, by comparing isotopologue patterns between saturated and MUFAs, we found that tumours contained a high proportion of MUFA not derived from de novo synthesis, suggesting that these may be taken up. This was unexpected, as it has been proposed that MUFAs are relatively scarce in in the brain microenvironment, making desaturation an essential metabolic requirement for brain metastasis^55^. Monitoring metabolism using ^13^C-imaging MS coupled to AIF thus provides insight into dynamic fatty acid provision in tumour and healthy tissues.

In addition, we used the palmitate isotopologue distribution to determine acetyl-CoA labelling in tumour tissue in a spatial manner. This revealed substantial spatial heterogeneity, possibly caused by differences in local availability of nutrients and oxygen in different tumour areas or by genetic heterogeneity of the implanted cells. The observed differences in palmitate isotopologue pattern, increase in *M* + 0 and shift toward isotopologues with lower mass were remarkably similar to those observed after exposure of cancer cells to experimental hypoxia or silencing of ACLY. It has been shown that ACLY promotes cell migration in glioblastoma through mechanisms involving histone modification^56^. Moreover, a recent spatial transcriptomics and proteomics study defined hypoxia as a major driver of long-range tissue organization in glioma^57^. It will be interesting to integrate ^13^C-SpaceM data with other spatial approaches to obtain deeper insight into mechanisms of metabolic heterogeneity in cancer.

Finally, we applied our methodology to analyse the metabolism of essential fatty acids in glioma. Notably, tumours showed evidence for elongation and desaturation of omega-6 essential fatty acids. This agrees with elevated expression enzymes responsible for the metabolism of omega-6 essential fatty acids observed in human low-grade glioma. Altered fatty acid metabolism is a hallmark of glioma^58^ and an increase in the ratio of omega-3 and omega-6 fatty acids has been described as a marker of aggressive disease^59^.

Our results present ^13^C-SpaceM as a methodology for spatial single-cell isotope tracing, able to monitor de novo fatty acid synthesis, composition of lipogenic acetyl-CoA and fatty acid uptake in heterogenous cell populations exposed to genetic and environmental perturbations. Through additional modifications of this approach, for example by applying different metabolic tracers or by selecting specific subsets of lipids using ion mobility separation, ^13^C-SpaceM could provide more specific information about lipid metabolism at single-cell resolution. Furthermore, with increased spatial resolution of MALDI imaging, and by complementing the analysis with single-cell segmentation in microscopy images, this method could be extended to single-cell isotope tracing in tissue sections. Finally, results can also be integrated with other spatial omics data, such as transcriptomics or proteomics, to obtain deeper insight into the metabolic programmes of cells and tissues.

### Limitations

Fatty acids analysed using the MALDI-AIF method applied here are derived from a subset of lipids that are effectively ionized in the negative mode. Some abundant lipid species, including phospatidylcholines, triglycerides and sphingomyelins, are underrepresented. Comparing data from MALDI-AIF with bulk MS after saponification revealed high concordance, indicating that fatty acids in the lipids sampled by MALDI-AIF are representative of the total lipidome. Notably, isotopologue distribution of fatty acids derived from de novo synthesis faithfully reflects labelling degree of the lipogenic acetyl-CoA pool at isotopic steady state independently of the identity of sampled lipids. However, the selectivity of the MALDI-AIF method has to be considered for the estimation of fatty acid uptake, as the proportion of fatty acids derived from uptake may differ depending on lipid class. In addition, our methodology can only provide information on relative substrate contribution to the lipogenic acetyl-CoA pool as opposed to absolute contribution, as the size of this pool cannot be quantified. Increased relative contribution from one substrate can either be caused by an increase in absolute contribution or by reduced contribution from another source. This limitation could be partially overcome by measuring relative contribution of several substrates in parallel. It also has to be considered that different fatty acid pools may reach isotopic steady state at different times after label addition. Accurate quantification of the relative contribution of de novo synthesis and uptake for different fatty acid species may require previous determination of time required to reach isotopic steady state. In the case of the in vivo model, isotopic steady state was likely not reached for fatty acids within the 48-h labelling period^38^. Thus, labelling data need to be interpreted under consideration of pool size data. Of note, this limitation does not apply to the assessment of substrate contribution to the lipogenic acetyl-CoA pool, as isotopic steady state for this pool is reached within a few hours of labelling^32^.

## Methods

### Cell culture for the normoxia-hypoxia model

Primary murine liver cancer cells derived from a Myc- and Akt-driven tumour model (*Myc*^OE^; *Akt*^Myr^; *Tp53*^−/−^)^15^ were a gift from D. Dauch (University of Tübingen). HEK293 cells were from ATCC and used at low passage. Cells were cultured in glucose-free DMEM (Sigma Aldrich) supplemented with 1 mM acetate, 2 mM glutamine and 10% dialysed FCS after addition of 25 mM of either ^12^C-glucose (Sigma) or U-^13^C-glucose (Cambridge Isotopes) in a 37 °C incubator with 5% CO_2_ either under normoxia (20% O_2_) or hypoxia (0.5% O_2_) for 72 h. The time point of 72 h was chosen to make sure that isotopic steady state for palmitate had been reached. Hypoxic conditions (0.5% O_2_) were induced in a hypoxia workstation (H35, Don Whitley). The cells cultured under hypoxic conditions were modified to express GFP as a marker. For co-plating experiments, normoxic and hypoxic cells were detached using trypsin, mixed using 10,000 cells of each condition, plated on the same glass slide and allowed to attach for 3 h before fixation.

### Western blot analysis

Cells were lysed in RIPA buffer (150 mM NaCl, 50 mM Tris, pH 8.0, 1% (*v*/*v*) NP-40, 0.5% (w/v) sodium deoxycholate and 0.1% (*w*/*v*) SDS) with protease and phosphatase inhibitors for 30 min and cleared by centrifugation. Proteins were quantified using BCA (Thermo Scientific). Proteins were separated by SDS–PAGE and blotted onto PVDF membrane (Immobilon), treated with blocking solution (5% BSA) and incubated with primary and secondary antibodies in 5% BSA. Signals were detected on a ChemiDoc (Bio-Rad). Antibodies used were anti-ACLY (Cell Signalling, 4331) at 1:100 dilution and anti-β-tubulin (Cell Signalling, 2148) at 1:5,000 dilution.

### ACLY knockdown

For the ACLY knockdown, we used the same primary murine liver cancer cells as in the hypoxia model. shRNA sequences targeting murine ACLY or non-targeting controls were cloned into LT3-GEPIR (Addgene); the shRNA sequences used were (TGCTGTTGACAGTGAGCGACCGCAGCAAAGATGTTCAGTATAGTGAAGCCACAGATGTATACTGAACATCTTTGCTGCGGCTGCCTACTGCCTCGGA), (TGCTGTTGACAGTGAGCGAACCAGTGTCTACTTATGTCAATAGTGAAGCCACAGATGTATTGACATAAGTAGACACTGGTCTGCCTACTGCCTCGGA) and (TGCTGTTGACAGTGAGCGCAGGAATTATAATGCTTATCTATAGTGAAGCCACAGATGTATAGATAAGCATTATAATTCCTATGCCTACTGCCTCGGA) for ACLYkd oligo 1, oligo 2 and non-targeting control, respectively. Lentiviral particles were produced in HEK293 cells after transient transfection of the packaging vectors psPAX.2 and pMD.G2 (Addgene, #12260 and #12259). After viral transduction, liver cancer cells were selected with puromycin and used at low passage. Induction of shRNA expression was achieved by treating cells with 1 µg ml^−1^ doxycycline (Sigma) for 72 h. For stable isotope tracing, cells were cultured in glucose-free DMEM supplemented with U^13^C_6_-glucose (Cambridge Isotope laboratory) and 1 mM acetate for 72 h.

### Analysis of fatty acids and lipids using LC–MS

For bulk LC–MS, cells were washed with cold 154 mM ammonium acetate, snap frozen in liquid nitrogen and collected in methanol:H_2_O (80:20, *v*/*v*) with added standards (for fatty acids, 10 µl 100 µM palmitate-2,2-D2 (Eurisotop, DLM-1153-0)/1 × 10^6^ cells For lipids (SPLASH LIPIDOMIX, Avanti Polar Lipids, 330707-1EA) 10 μl per sample). Subsequently, 30 μl 0.2 M HCl, 2 × 100 μl CHCl_3_ and 2 × 100 μl H_2_O was then added with vortexing in between. The suspension was centrifuged at 16,000*g* for 5 min at room temperature, the lower lipid phase was then washed with synthetic polar phase (CH_3_Cl:methanol:H_2_O, 58:33:8, *v*/*v*/*v*) and evaporated to dryness under N_2_ at 45 °C. For lipidomics the samples were resuspended and subjected to LC–MS analysis. For fatty acid analysis lipid extract was saponified by resuspension in methanol:H_2_O (80:20, *v*/*v*) containing 0.3 M KOH, heating at 80 °C for 1 h and washed twice with 0.5 ml hexane. After addition of 50 μl formic acid, fatty acids were subsequently extracted twice with 0.5 ml hexane and evaporated to dryness under N_2_ at 45 °C. For LC–MS analysis the fatty acids, were dissolved in 100 μl isopropanol and 5 μl of each sample was applied to a C8 column (Accucore C8 column, 2.6-µm particle size, 50 × 2.1 mm, Thermo Fisher Scientific) at 40 °C, with mobile phase A consisting of 0.1% formic acid in CH_3_CN:H_2_O (10:90, *v*/*v*) and solvent B consisting of 0.1% formic acid in CH_3_CN:H_2_O (90:10, *v*/*v*). The flow rate was maintained at 350 μl min^−1^ and eluent was directed to the ESI source of a mass spectrometer from 3 min to 27 min after sample injection. MS analysis was performed on a Q Exactive Plus Orbitrap mass spectrometer (Thermo Fisher Scientific) applying the following settings: sheath gas, 30; spray voltage, 2.6 kV. Capillary temperature 320 °C, aux gas heater temperature: 120 °C and S-lens voltage was 55. A full scan range from 150 to 460 (*m*/*z*) in negative ion mode was used. The resolution was set at 70,000. The maximum injection time was 100 ms with an AGC target of 1 × 10^6^. For lipidomic analysis, lipids were separated on a C8 column (Accucore C8 column, 2.6 µm particle size, 50 × 2.1 mm, Thermo Fisher Scientific) mounted on an Ulitmate 3000 HPLC (Thermo Fisher Scientific) and heated to 40 °C. The mobile phase buffer A consisted of 0.1% formic acid in CH_3_CN:H_2_O (10:90, *v*/*v*) and buffer B consisted of 0.1% formic acid in CH_3_CN:IPOH:H_2_O (45:45:10, *v*/*v*/*v*). After injection of a 3-µl lipid sample, 20% solvent B was maintained for 2 min, followed by a linear increase to 99.5% B within 5 min, which was maintained for 27 min. After returning to 20% B within 1 min, the column was re-equilibrated at 20% B for 5 min, resulting in a total run time of 40 min. The flow rate was maintained at 350 µl min^−1^ and the eluent was directed to the ESI source of the QE Plus from 2 to 35 min. MS analysis was performed on a Q Exactive Plus mass spectrometer (Thermo Fisher Scientific) applying the following settings: scan settings, scan range of 200–1,600 *m*/*z* in full MS mode with switching polarities (neg/pos) and data-dependent fragmentation; resolution of 70,000, AGC target of 1E6; max. injection time of 50 ms. HESI source parameters: sheath gas of 30; aux gas of 10; sweep gas of 3; spray voltage of 2.5 kV; capillary temperature of 320 °C; S-lens RF level of 55.0; aux gas heater temperature of 55 °C; fragmentation settings: resolution of 17,500; AGC target of 1E5; and max. injection time of 50 ms. Peaks corresponding to the calculated fatty acid or lipid masses (± 5 ppm) were integrated using El-Maven (https://resources.elucidata.io/elmaven) and correction for natural ^13^C isotopic abundance was conducted using IsoCorrectoR^60^.

### Analysis of fatty acid distribution and isotopologue distribution by direct infusion MS

For the analysis of fatty acid distribution and isotopologue distribution using direct infusion, saponified fatty acids, collected as described above, were resuspended in 50:50 solution A and B (A, H_2_O:ACN:isopropanol 2:10:88 and B, H_2_O:ACN 60:40 both with 10 mM ammonium acetate). Samples were injected by direct infusion at 20 μl min^−1^ for 2 min whereas spectra were acquired for a total of 8 min (3 min before and after injection to establish baseline) using the following scan parameters: full MS scan range of 69–1,000 *m*/*z*, AGC target 1 × 10^6^, resolution of 70,000 in negative mode, maximum injection time 100 ms. HESI source parameters were as follows, sheath gas flow rate 6, spray voltage 3.20 kV, S-lens RF level 50 and aux gas temperature 120 °C. For AIF, samples were not saponified and lipid extract was resuspended the same as described above. All parameters were the same except that the scan type was AIF, precursor masses were collected in the range of 600–1,000 *m*/*z*, the scan range was 200–350 *m*/*z*, fragmentation was carried out using CE of 50 and the maximum injection time was 200 ms.

### Microscopy

After cell culturing, the cells were washed with PBS, fixed using Histofix (Roth, 87.3) for 10 min, stained using 4,6-diamidino-2-phenylindole (DAPI), washed 3× in PBS and then desiccated in a Lab Companion Cabinet Vacuum Desiccator for 30 min at room temperature and −0.08 MPa. Pre-MALDI brightfield and fluorescent microscopy (620 and 460 nm) images were obtained with a DS-Qi2 camera (Nikon Instruments) with a Plan Fluor ×10 (numerical aperture of 0.30) objective (Nikon Instruments) mounted on a Ti-E inverted microscope (Nikon Instruments). The pixel size was 0.64 μm. For the hypoxia experiment, additional pre-desiccation microscopy images were collected. Rigid registration of pre-desiccation and post-desiccation microscopy images was performed using the Affinder plugin in Napari (https://www.napari-hub.org/plugins/affinder). The cells were imaged in brightfield microscopy after MALDI imaging, using the same microscopy setup and parameters.

### Microscopy image analysis

Cells in the brightfield channel of pre-MALDI microscopy were segmented using a custom Cellpose model^61^ trained on manually annotated fragments of images. Since the primary cell culture contains cells of varying size, segmentation for the largest cells had to be manually corrected. Ablation marks were detected in the post- MALDI microscopy by manually fitting a grid of circular shapes. Registration of the pre- and post-MALDI images was carried out using the SpaceM software tool as previously published^31^.

### MALDI imaging of single-cell samples

The MALDI matrix 1,5-diaminonaphthalene (DAN) was applied to a surface density of 3 μg μm^−2^ immediately before MALDI analysis using a TM sprayer (HTX Technologies). Atmospheric pressure MALDI imaging was performed using an AP-SMALDI5 ion source (TransMIT) coupled to a Q Exactive Plus Orbitrap mass spectrometer (Thermo Fisher Scientific). Raster pitch was 10 × 10 μm, with the laser attenuator angle set to 33°. The MS method used was set up as AIF in negative ion mode, with an isolation range of 600–1,000 *m*/*z* and a scan range of 100–400 *m*/*z* at 140,000 resolution with 500 ms maximum ion injection time. Fragmentation was performed using higher-energy collisional dissociation at a normalized collision energy (NCE) of 25. This energy level was determined by a manual stepped collision energy experiment, where we saw no improvement in fatty acid signal/noise by going as high as 45 NCE. Only one collision energy can be used per ion injection and one ion injection was performed per pixel. Thus, data acquisition for this type of experiment is limited to one collision energy.

### SpaceM analysis

The ^13^C-SpaceM method is based on SpaceM described in detail previously^31^. The key differences are in using AIF (see ‘MALDI imaging of single-cell samples’ section) and processing obtained profiles (see the respective section of Methods). In brief, SpaceM integrates microscopy images and the MALDI images by detecting the MALDI ablation marks, overlaying them with the segmented cells and performing ablation marks-cells deconvolution by applying a mathematical formula. This results in single-cell profiles of molecules detected by the MALDI imaging MS.

#### Constructing single-cell isotopologue profiles

MALDI images for unlabelled samples were annotated with METASPACE^62^ at 5% false discovery rate to determine the most abundant detected fatty acids. For each fatty acid, intensities corresponding to theoretical isotopologue peak masses were extracted. Raw intensities were normalized for natural isotope abundance using IsoCor^63^, after which every isotopologue distribution was normalized by its sum. Ablation marks with a total raw intensity of less than 200 for a given fatty acid were removed from the analysis. Ablation marks which had at least 30% overlap with the cell mask were considered intracellular. After normalizing spectra in each pixel, the median of values in the intracellular ablation marks was assigned as a single-cell readout for each isotopologue peak. The resulting single-cell isotopologue distribution for every fatty acid was normalized by its sum again. Scanpy v.1.8.1 (ref. ^64^) was used for all single-cell data analysis.

#### Calculation of single-cell features

For each well of a multi-well slide, the median intensity of the GFP channel outside the cell masks was subtracted from the image and the logarithm of the maximum GFP intensity inside the cell mask was used to characterize single-cell GFP signal. Both in normoxia/hypoxia and in ACLYkd experiments, the 95th percentile of the GFP signal in the GFP-negative wells was used as a threshold to assign cells to either GFP-positive or GFP-negative state.

In addition to the normalized isotopologue peak intensities, a binomial model for the fatty acid synthesis described previously^32^ was used. Fatty acids are synthesized by randomly taking two-carbon monomers from the cytosolic acetyl-CoA pool. Therefore, if the fraction of labelled monomers equals *p*, the probability that a given fatty acid has 2*i* labelled carbons can be described with the following model:

$${P}_{\rm{binom}}\left(k\right)={{n}\choose{k}}{p}^{k}{\left(1-p\right)}^{n-k},\,k=0,1,\ldots,n$$

$${I}_{0}=\mathrm{{Uptake}}+\left(1-\mathrm{{uptake}}\right)\times {P}_{{\mathrm{binom}}}(0)$$

$${I}_{i}=\left(1-\mathrm{{uptake}}\right)\times {P}_{\mathrm{{binom}}}(i),\,i=1,2,\ldots,n$$

where uptake is the fraction of the unlabelled FA directly taken from the medium, *p* is the labelling degree of cytosolic acetyl-CoA pool and *n* is the number of acetyl-CoA molecules used for the synthesis (number of carbons in the fatty acid/2). Longer fatty acids can be synthesized both using labelled palmitate made by the cell de novo and unlabelled palmitate; therefore, we used a modified model for stearate and oleate (C18):

$${I}_{0}=\mathrm{{Uptake}}+\left(1-\mathrm{{uptake}}\right)\times {P}_{\mathrm{{binom}}}\left(0\right)$$

$${I}_{1}=\left(1-{\rm{uptake}}\right)\times \left({\rm{uptak}{e}}_{C16}+\left(1-{\rm{uptak}{e}}_{C16}\right)\times {P}_{\rm{binom}}\left(1\right)\right)$$

$${I}_{i}=\left(1-\rm{uptake}\right)\times \left(1-\rm{uptak}{e}_{C16}\right)\times {P}_{\rm{binom}}(i),\,i=2,3,\ldots,n$$

where uptake_C16_ is the fraction of palmitate (C16) taken from the medium.

Fitting a binomial model to the single-cell isotopologue distributions allows summarizing them as two numbers: uptake, which characterizes the fraction of the fatty acid which was not synthesized de novo but directly taken from the medium and acetyl-CoA pool labelling degree *p*, which describes relative contribution of the labelled substrate to the fatty acid synthesis compared with other carbon substrates consumed by the cell. The limitation of this method is that if overall substrate usage is very low, such as in the hypoxia/normoxia experiment, it becomes impossible to reliably fit a binomial distribution and estimate the uptake fraction, therefore it was only used for the ACLYkd experiment data analysis.

### Orthotopic glioma mouse model

Tissue sections were prepared from mouse brains used for a previous study^38^. The experiments were approved by the Institutional Animal Care and Use Committee at Washington University (assurance no. A338101, protocol 19-0930 and 22-0304) and were performed in accordance with the recommendations in the Guide for the Care and Use of Laboratory Animals of the National Institutes of Health (NIH). In brief, murine glioma cells (GL261-RFP, transduced with IDH1 R132H) were implanted into female mice (C57BL/6J, 8 weeks old). After 8 days, mice were fed a liquid diet containing unlabelled glucose or U-^13^C-labelled glucose (Cambridge Isotope Laboratories) ad libitum for 48 h as previously described^38,53^. Brains were embedded in 5% wt. carboxymethyl cellulose in water and stored at −80 °C. The 10-µm thick sections were collected on Superfrost Plus slides (Thermo Fisher Scientific), dried under vacuum, stored at −80 °C and shipped on dry-ice. Serial 10-µm thick tissue sections were mounted on Superfrost Plus slides in Fluoroshield mounting medium with DAPI (aqueous, Abcam) and used for fluorescence microscopy to verify tumour location on a Leica DMi8 Thunder Imager (RFP, excitation 540–580, DC 585, emission 592–668 and exposure 1.3 s; DAPI, excitation 375–435, DC 455, emission 450–490, exposure 59 ms)^38^.

### MALDI tissue imaging

Imaging of brain sections was performed using the same sample preparation method and instrumentation described for single cells. For each whole-brain section, one image of the tumour and immediate environment was first acquired at 10 × 10 µm pitch, followed by an image of the rest of the tissue section at 50 µm pitch. The laser attenuation angle was set to 32°, the isolation range was 600–1,600 *m*/*z*, product scan range 100–600 *m*/*z* and the NCE set to 30.

### Data analysis for tissue imaging

Each tissue image was converted to the imzML format and annotated with METASPACE^62^ in the same way as the single-cell data. Compared with processing data from the cells, no cell segmentation was performed and all data analysis was performed on single MALDI pixels (with the pitch of 10 µm within the tumour area and with 50 µm within the rest of the tissue section). The same normalization and AcCoA pool labelling degree modelling was applied as for the cells and figures were exported as spatial heatmaps using the same custom script as for single-cell data

### Statistics and reproducibility

No statistical method was used to predetermine sample size. No statistical method was used to determine sample size. Single-cell experiments were performed on a single biological replicate, covering between 900–2,000 cells per condition. The variance was a result of different cell densities for the same imaged area. The mouse experiments were performed on four different mice, plus one animal without the isotope labelling treatment for use as control. This sample number was determined by sample availability from a previous study and no sample size dependent statistics were used. There were no relevant sample groups in this study for which randomization was applicable. One out of six technical replicates for the co-cultured cells (wild-type and ACLYkd oligo 2) was excluded as it did not pass quality control. Investigators were not blinded to allocation during experiments and outcome assessment. No animals were excluded from the analysis.

### Reporting summary

Further information on research design is available in the Nature Portfolio Reporting Summary linked to this article.

## Supplementary information

Reporting Summary

Supplementary Table 1 List of masses detected in the murine HCC (*Myc*^OE^; *Akt*^Myr^; *Tp53*^−/−^) cell line using single-cell imaging MS (600–1,000 *m*/*z*) and automatically annotated using METASPACE.

Supplementary Table 2 List of lipids identified in the murine HCC (*Myc*^OE^; *Akt*^Myr^; *Tp53*^−/−^) cell line using LC–MS/MS.

## Source data

Source Data Extended Data Fig. 3 Unprocessed western blots for Extended Data Fig. 3a.

## Data Availability

All MALDI data in this paper are available at https://metaspace2020.eu/project/buglakova-2024. Raw data and the final single-cell data are available through accession no. S-BSST1436. Source data are provided with this paper.

## Code availability

The code for producing the results and figures is available at https://github.com/Buglakova/13C-SpaceM.
