# Supplementary material for: Spatial single-cell isotope tracing reveals heterogeneity of de novo fatty acid synthesis in cancer
Source: Nat Metab. 2024 Sep 9;6(9):1695–711. doi: 10.1038/s42255-024-01118-4 (PMC11422168; doi:10.1038/s42255-024-01118-4)
Supplement: Supplementary file 1 — Reporting Summary [file 42255_2024_1118_MOESM1_ESM.pdf]

nature portfolio

Corresponding author(s): Theodore Alexandrov

Last updated by author(s): Feb 6, 2024

Reporting Summary

Nature Portfolio wishes to improve the reproducibility of the work that we publish. This form provides structure for consistency and transparency in reporting. For further information on Nature Portfolio policies, see our Editorial Policies and the Editorial Policy Checklist.

Statistics

For all statistical analyses, confirm that the following items are present in the figure legend, table legend, main text, or Methods section.

n/a	Confirmed	
☐	☒	The exact sample size (*n*) for each experimental group/condition, given as a discrete number and unit of measurement
☐	☒	A statement on whether measurements were taken from distinct samples or whether the same sample was measured repeatedly
☐	☒	The statistical test(s) used AND whether they are one- or two-sided<br>*Only common tests should be described solely by name; describe more complex techniques in the Methods section.*
☒	☐	A description of all covariates tested
☒	☐	A description of any assumptions or corrections, such as tests of normality and adjustment for multiple comparisons
☒	☐	A full description of the statistical parameters including central tendency (e.g. means) or other basic estimates (e.g. regression coefficient) AND variation (e.g. standard deviation) or associated estimates of uncertainty (e.g. confidence intervals)
☐	☒	For null hypothesis testing, the test statistic (e.g. *F*, *t*, *r*) with confidence intervals, effect sizes, degrees of freedom and *P* value noted<br>*Give P values as exact values whenever suitable.*
☒	☐	For Bayesian analysis, information on the choice of priors and Markov chain Monte Carlo settings
☒	☐	For hierarchical and complex designs, identification of the appropriate level for tests and full reporting of outcomes
☒	☐	Estimates of effect sizes (e.g. Cohen's *d*, Pearson's *r*), indicating how they were calculated

Our web collection on statistics for biologists contains articles on many of the points above.

Software and code

Policy information about availability of computer code

Data collection	The data was collected using the commercial software provided by ThermoFisher Scientific (mass spectrometry vendor) and TransMIT (MALDI-source vendor).
Data analysis	The following open Python libraries were used: isocor v2.2.0, pyimzml v1.5.1, scanpy v1.8, matplotlib v3.6.2, pandas=1.2.4, scikit-learn v1.0.2, scikit-image v0.19.2.The code for producing the results and figures is available at https://github.com/Buglakova/13C-SpaceM.

For manuscripts utilizing custom algorithms or software that are central to the research but not yet described in published literature, software must be made available to editors and reviewers. We strongly encourage code deposition in a community repository (e.g. GitHub). See the Nature Portfolio guidelines for submitting code & software for further information.

Data

Policy information about availability of data

All manuscripts must include a data availability statement. This statement should provide the following information, where applicable:

- Accession codes, unique identifiers, or web links for publicly available datasets
- A description of any restrictions on data availability
- For clinical datasets or third party data, please ensure that the statement adheres to our policy

All MALDI data in this paper is available at https://metaspace2020.eu/project/buglakova-2023. Raw microscopy, cell segmentation and single-cell isotope tracing

data are available on BioStudies under accession number S-BSST1436
Data for expression analysis in low grade glioma are from TCGA and GTEx

Research involving human participants, their data, or biological material

Policy information about studies with human participants or human data. See also policy information about sex, gender (identity/presentation), and sexual orientation and race, ethnicity and racism.

Reporting on sex and gender	N/A
Reporting on race, ethnicity, or other socially relevant groupings	N/A
Population characteristics	N/A
Recruitment	N/A
Ethics oversight	N/A

Note that full information on the approval of the study protocol must also be provided in the manuscript.

Field-specific reporting

Please select the one below that is the best fit for your research. If you are not sure, read the appropriate sections before making your selection.

☒ Life sciences ☐ Behavioural & social sciences ☐ Ecological, evolutionary & environmental sciences

For a reference copy of the document with all sections, see nature.com/documents/nr-reporting-summary-flat.pdf

Life sciences study design

All studies must disclose on these points even when the disclosure is negative.

Sample size	No statistical method was used to determine sample size. Single-cell experiments were performed on a single biological replicate, covering between 900-2000 cells per condition. The variance was a result of different cell densities for the same imaged area. The mouse experiments were performed on four different mice, plus one mouse sample without the isotope labelling treatment for use as control. This sample number was determined by sample availability from a previous study, and no sample size dependent statistics were used.
Data exclusions	One out of six technical replicates for the co-cultured cells (wildtype and ACLY.kd Oligo 2) was excluded as it did not pass QC. The MALDI data are included in the raw data repository, but were excluded from figures. The total number of cells from this condition was deemed sufficient (1716 total).
Replication	Each single-cell experiment was technically replicated at least three times. No biological replicates were deemed necessary. Analysis of isotopically labelled mouse brains was successfully performed in four biological replicates.
Randomization	There were no relevant sample groups in this study for which randomization was applicable.
Blinding	There were no relevant sample groups in this study for which blinding was applicable.

Reporting for specific materials, systems and methods

We require information from authors about some types of materials, experimental systems and methods used in many studies. Here, indicate whether each material, system or method listed is relevant to your study. If you are not sure if a list item applies to your research, read the appropriate section before selecting a response.

Materials & experimental systems

n/a	Involved in the study	
☐	☒	Antibodies
☐	☒	Eukaryotic cell lines
☒	☐	Palaeontology and archaeology
☐	☒	Animals and other organisms
☒	☐	Clinical data
☒	☐	Dual use research of concern
☒	☐	Plants

Methods

n/a	Involved in the study	
☒	☐	ChIP-seq
☒	☐	Flow cytometry
☒	☐	MRI-based neuroimaging

Antibodies

Antibodies used	For western blotting anti ATP-Citrate lyase (ACLY) (Cell signaling #4332) 1/1000 in 5%BSA and anti Tubulin (Cell signaling #2148)1/5000 in 5%BSA
Validation	For Western Blotting: All antibodies were obtained from trusted sources and were confirmed to detect proteins of the correct size. Specific signals for ACLY were confirmed by gene silencing.

Eukaryotic cell lines

Policy information about cell lines and Sex and Gender in Research

Cell line source(s)	Primary murine liver cancer cells derived from a Myc- and Akt-driven tumor model (MycOE; AktMyr; Tp53–/–) 15 were a gift from Daniel Dauch (University of Tübingen).<br>HEK293T were obtained from ATCC.
Authentication	All cell lines were obtained from a trusted source and used at low passage.<br>HEK293T were authenticated by ATCC.
Mycoplasma contamination	All cell lines were regularly tested for the absence of mycoplasma contamination and found to be negative.
Commonly misidentified lines<br>(See ICLAC register)	No commonly misidentified lines listed in ICLAC register were used in this study

Animals and other research organisms

Policy information about studies involving animals; ARRIVE guidelines recommended for reporting animal research, and Sex and Gender in Research

Laboratory animals	Female mice (C57BL/6J, eight weeks old) were obtained from Jackson Laboratory. Mice were housed with an ambient temperature of 22 degrees Celsius, a relative humidity of 55%, and a 12-h light/dark cycle.
Wild animals	No wild animals were used in this study.
Reporting on sex	Sex was not considered in study design.
Field-collected samples	No field collected samples were used in this study.
Ethics oversight	Animal experiments were approved by the Institutional Animal Care and Use Committee at Washington University (assurance number A338101, protocol 19-0930 and 22-0304) and were performed in accordance with the recommendations in the Guide for the Care and Use of Laboratory Animals of the NIH.

Note that full information on the approval of the study protocol must also be provided in the manuscript.

Plants

Seed stocks	*Report on the source of all seed stocks or other plant material used. If applicable, state the seed stock centre and catalogue number. If plant specimens were collected from the field, describe the collection location, date and sampling procedures.*
Novel plant genotypes	*Describe the methods by which all novel plant genotypes were produced. This includes those generated by transgenic approaches, gene editing, chemical/radiation-based mutagenesis and hybridization. For transgenic lines, describe the transformation method, the number of independent lines analyzed and the generation upon which experiments were performed. For gene-edited lines, describe the editor used, the endogenous sequence targeted for editing, the targeting guide RNA sequence (if applicable) and how the editor was applied.*
Authentication	*Describe any authentication procedures for each seed stock used or novel genotype generated. Describe any experiments used to assess the effect of a mutation and, where applicable, how potential secondary effects (e.g. second site T-DNA insertions, mosiacism, off-target gene editing) were examined.*
