# Supplementary figures and images for: Spatial single-cell isotope tracing reveals heterogeneity of de novo fatty acid synthesis in cancer
Source: Nat Metab. 2024 Sep 9;6(9):1695–711. doi: 10.1038/s42255-024-01118-4 (PMC11422168; doi:10.1038/s42255-024-01118-4)

**Uncropped WB images:**

Extended Data Figure 3a (ACLY)

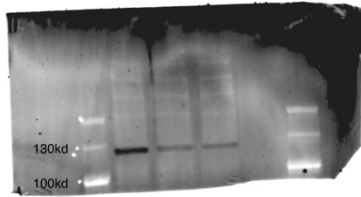

Extended Data Figure 3a (Tubulin)

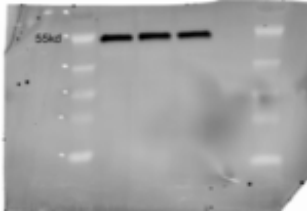

Supplement: Supplementary file 4 — Unprocessed western blots for Extended Data Fig. 3a. [file 42255_2024_1118_MOESM4_ESM.pdf]
